# Substrate type and termite discovery shape decomposition at the dry end of an African savanna rainfall gradient

**DOI:** 10.1007/s00442-026-05960-z

**Published:** 2026-08-28

**Authors:** Katherine Bunney, Mark Robertson, Paul Eggleton, Sally Archibald, Catherine Parr

**Affiliations:** 1 Department of Zoology & Entomology, University of Pretoria, Pretoria, South Africa; 2 Elephants Alive, Ekuthuleni Shareblock Ltd, Hoedspruit, South Africa; 3 Entomology Department, The Soil Biodiversity Group, The Natural History Museum, London, UK; 4 School of Animal, Plant and Environmental Sciences, University of Witwatersrand, Johannesburg, 2000 Republic of South Africa; 5 School of Environmental Sciences, University of Liverpool, Liverpool, UK

**Keywords:** Savannas, South Africa, Semi-arid, Decomposition, Termites, Microbes, Dung, Wood, Grass

## Abstract

Decomposition is the process by which dead plant biomass is recycled and made available again for uptake by other plants. It is largely mediated by microbes and soil invertebrates. Global decomposition studies have demonstrated that decomposition is primarily temperature-driven with rainfall playing a secondary role, although to date, all global decomposition studies have used a single substrate to measure decomposition meaning differences with substrate type may be missed. Here we explored the decomposition of wood, dung and grass along a semi-arid rainfall gradient (380–650 mm/year) at three savanna sites in north-eastern South Africa. As the three sites experience a similar temperature range, we effectively controlled for temperature allowing us to explore the effect of rainfall in isolation. We used decomposition bags - with and without termite access - and measured mass loss after set time intervals. We predicted that the absolute decomposition of all three substrates would increase along the rainfall gradient. This assumption held for dung and grass, but we found the reverse for wood. Our study highlights the importance of considering multiple substrates as decomposition patterns and dominant agents can vary. Unexpectedly low wood decomposition at our wettest site suggests that the amount of dead wood available, and therefore probability of discovery by termites, may be an important factor explaining findings from decomposition studies. Resource availability and termite discovery is a neglected subject within decomposition studies and warrants further investigation.

**Supplementary Information:**

The online version contains supplementary material available at https://doi.org/10.1007/s00442-026-05960-z.

## Introduction

Decomposition, the process by which the stock of nutrients and carbon in dead plants is recycled, converting dead organic matter to plant available nutrients (Pausas and Bond 2020; Griffiths et al. 2021), and is primarily brought about by biotic agents: fungi, bacteria, and soil/litter invertebrates. The dominant conceptual model of decomposition suggests that the primary controls of the rate of decomposition are climate, litter quality (substrate attributes) and decomposer organisms. These controls have traditionally been thought to function hierarchically, with climate and litter quality dominant at broad scales, and decomposers operating only as a secondary small-scale control (although see Bradford et al. 2017). Climate specifically, temperature and rainfall, strongly determine the rate of decomposition (Lavelle et al. 1993; Couteaûx et al. 1995; Aerts 1997) as microbial (fungal and bacterial) decomposition is positively affected by both temperature and moisture. However, under extremes of temperature and moisture, decomposition takes place more slowly as microbial communities are sensitive to such extremes and become inactive (Couteaûx et al. 1995). Second, the process of decomposition is co-dominated by the chemical and physical composition of the substrate (litter quality), which can have a profound effect on the abundance and activity of decomposer communities resulting in different rates of decomposition (Taylor et al. 1989; Aerts 1997; Mackensen et al. 2003; Cornwell et al. 2008).

In terms of decomposition agents, there has, historically, been a focus on microbial decomposition (Griffiths et al. 2021), possibly because most of these studies have been conducted principally in temperate regions. This microbial model of decomposition has informed earth system models (Meentemeyer, CENTURY and the GEM model–Meentemeyer 1978; Parton et al. 1993; Rastetter et al. 1991) which predict increased decomposition rates with increased temperature and rainfall. More recent studies have focussed on the role invertebrates play in decomposition (e.g. Wall et al. 2008; Griffiths et al. 2019; Seibold et al. 2021; Zanne et al. 2022). For example, a global decomposition study found that insects were responsible for 29% of deadwood turnover across forest systems (Seibold et al. 2021), while in the tropics and subtropics, termites can be the dominant invertebrate decomposer of wood (Griffiths et al. 2021).

Termites dominate the soil in the Tropics, comprising 40–60% of total soil macrofaunal biomass (Potapov et al. 2022), with up to 12 million individuals per hectare (Dahlsjö et al. 2014). In African savannas, termite biomass is immense (estimated between 70 and 110 kg ha^-1^; Woods and Sands 1978, Moe et al. 2009) and comparable to that of both ungulates and megaherbivores (< 110 kg ha^-1^, Owen-Smith 1988). Termites feed on a wide range of dead plant material at various stages of decay (Holt and Lepage 2000), and can make use of this variety of food substrates owing to their intimate association with microbial symbionts either in their gut or, in the case of Macrotermitinae (the fungus-growing termites), in their mounds (Bignell 2016; Holt and Lepage 2000). Additionally, through the building of nest structures, tunnels, and soil sheeting, termites control their environment by maintaining a constant humidity within these structures (Jouquet et al. 2011). This allows them to remain active in arid environments and during times of diminished rainfall, where other soil macrofauna and microbes have low abundances or are absent (Jouquet et al. 2011). Fungus-growing termites, in particular, demonstrate notable resistance to aridity and recent theoretical modelling work indicates they may even confer some buffering to drought/desertification in dry African savannas (Bonachela et al. 2015). Furthermore, a second global decomposition study across all biomes found that termite-mediated wood decomposition peaks in tropical semi-arid areas (Zanne et al. 2022); this finding suggests that termites can, to some extent, decouple decomposition from the precipitation.

Savannas make up roughly 50% of the African continent (15.1 million km^2^; Grace et al. 2006). The decomposition of its major substrates - wood, grass, and dung - have been investigated to varying degrees with inconsistent findings. Wood decomposition has been explored across a wide rainfall gradient: the arid savannas of Tsavo National Park in Kenya: 200–500 mm p.a. - to a wet Southern Guinean savanna in Nigeria: 1175 mm p.a (Buxton 1981; Collins 1981; Schuurman 2006). However, only one study by Buxton (1981) explicitly explores wood decomposition across a rainfall gradient (200–500 mm p.a. - semi-arid savannas in Kenya). He found that wood decomposition by termites increased with increasing rainfall. Dung decomposition has received less attention (Freymann et al. 2008). A study by Veldhuis et al. (2017) is the only study to have quantified dung decomposition along a rainfall gradient (500–900 mm p.a. in Hluhluwe-iMfolozi Park, South Africa). They found that as rainfall increased, dung decomposition decreased, though they did not separate out the contribution of termites versus microbes. Grass decomposition in African savannas has been explored on a few occasions with conflicting results. Davies et al. (2015) found that across four distinct savanna types (ranging from 450 mm p.a. to 900 mm p.a.) the rate of decomposition increased under warmer and wetter conditions. In contrast, Buitenwerf et al. (2011) and Gosling et al. (2016) both found that across the rainfall gradient within Hluhluwe-iMfolozi Park (630–1000 mm p.a.) grass decomposition decreased with increasing rainfall. These differences may be because first, temperature and rainfall can be confounding variables (Chevalier and Chase 2016), and second, the studies differ in methods, collection intervals and substrates which makes comparison challenging.

Furthermore, the lack of published studies that examine more than a single substrate along the same rainfall gradient means that the relative importance of the agents of decomposition (microbes versus termites) to these different substrates remains poorly understood. In the face of global climate change, it is necessary that we understand how changing rainfall and temperature regimes will affect the flow of carbon in savanna systems. Disentangling the relative role of termites versus microbes to the decomposition of the different savanna substrates will help improve the accuracy of current earth system models.

In this study, we quantified wood, dung, and grass decomposition at three savanna sites across a semi-arid rainfall gradient in South Africa (Nwanedi Nature Reserve – 380 mm p.a.; Satara – 550 mm p.a. and Wits Rural Facility – 650 mm p.a.) in order to determine (1) how the absolute decomposition rate changes across the rainfall gradient, (2) how the relative contribution of decomposition agents varies, and (3) whether the termite community composition differs in both the genera and feeding groups across this same rainfall gradient.

We predicted that:

First, absolute decomposition would increase with rainfall because (a) microbial decomposition would likely increase with rainfall owing to the dependence of microbes on moisture for the process of decomposition and (b) termite decomposition would remain relatively constant across the gradient owing to their ability to engineer their environment. We expected the increase in absolute decomposition with rainfall to be especially pronounced for dung and grass as the greater surface area to volume ratio supports increased microbial decomposition.

Second, we predicted a greater relative contribution of microbial decomposition at higher rainfall with termites dominating decomposition at lower rainfall, where moisture would likely constrain microbial activity. Again we expected this pattern to be more pronounced for grass and dung.

Third, we expected to see differences in termite community composition along the rainfall gradient owing to observable changes in the savanna structure. For example, we expected a greater proportion of grass-feeding termites in both the 550 mm and 650 mm site than in the 380 mm site, since the biomass of grass at the higher rainfall sites is far greater than that at the lowest rainfall site where the grass layer is poorly developed and interspersed with herbs.

## Methods

### Study sites

We sampled in three savanna sites differing in mean annual rainfall: Wits Rural Facility (650 mm p.a.), Satara (550 mm p.a.) and Nwanedi Nature Reserve (380 mm p.a.). The rainy season across all three sites runs from December to March (consecutive months that cumulatively received 70% of the annual rainfall; Fick and Hijmans 2017 – approx. 120 days). Rainfall during the study period (January 2019 to December 2019) was consistent with the long-term means at each site (see Figure S1a). Rainfall data for each site were obtained directly from on-site weather stations operated at Wits Rural Facility, Satara (Kruger National Park), and Nwanedi Nature Reserve. These records reflect measured totals for the 2019 study period. By contrast, mean minimum and maximum temperatures were derived from WorldClim 2 (1-km resolution; Fick and Hijmans 2017) using site coordinates. All three sites have very similar mean minimum and maximum temperatures (Figure S1b). Because these temperature values represent long-term interpolated climatologies (1970–2000), they do not capture short-term or microclimatic variation (e.g. near-surface or canopy-shaded conditions). We therefore interpret climatic associations with decomposition cautiously, emphasising relative rather than absolute comparisons.

**Wits Rural Facility (WRF)** is in Limpopo Province, South Africa (24°33′58″S, 31°05′53″E). The site is at an elevation of 580 m a.s.l. and the vegetation type is classified as Granite Lowveld Savanna (Mucina and Rutherford 2006). The dominant tree species are *Terminalia sericea*, *Combretum collinum*, *Sclerocarya birrea* and *Dichrostachys cinerea*. Dominant grasses include: *Panicum maximum*, *Heteropogon contortus* and *Themeda triandra*. The Mpuluzi granites form the major basement geology of the area. The granites weather into sandy soils in the uplands and clayey soils with a high sodium content in the lowlands (Mucina and Rutherford 2006). The mammal density is estimated to be 0.4 animals/ha.

**Satara** (**SAT**) is in the central region of Kruger National Park, South Africa (24°23′35″S, 31°46′52″E). The site is at an approximate elevation of 300 m a.s.l. and the vegetation type is classified as Tshokwane Hlane Basalt Lowveld (Mucina and Rutherford 2006). The dominant tree species are *Senegalia (Acacia) nigrescens*,* Dichrostachys cinerea* and *Sclerocarya birrea*. Dominant grasses include *Bothriochloa radicans*, *Digitaria eriantha*, *Panicum coloratum*, *Urochloa mosambicensis*, and *Themeda triandra* (Knapp et al. 2012 in Donaldson et al. 2018). The Letaba Formation basalts form the basement geology of the area. The basalts weather into clay soils that are nutrient rich (Venter et al. 2003; February and Higgins 2010).

**Nwanedi Nature Reserve (NNR)** is in the Limpopo Province, South Africa (22°36′36″S, 30°22′41″E). The site is at an elevation of ~ 520 m a.s.l. and the vegetation type is classified as Limpopo Ridge Bushveld savanna on the rocky ridges (Mucina and Rutherford 2006). Dominant tree species are *Colophospermum mopane*, *Combretum apiculatum*, *Grewia flava* and *Sesamothamnus lugardii* (Mucina and Rutherford 2006). The herbaceous layer is poorly developed and dominated by *Schmidtia pappophoroides*. The area is underlain by both the sandstones of the Clarens Formation and basalts of the Karoo Supergroup (Brandl 1981). These give rise to shallow gravel and sand to calcium-rich clay soils (Mucina and Rutherford 2006). The mammal density is estimated to be very low, 0.09 animals/ha (calculated based on the mammal survey results in Ledet 2012–2017).

Although the three sites span a clear rainfall gradient, rainfall covaries with other environmental variables such as vegetation structure, soil characteristics, and herbivore activity. Consequently, the gradient should be viewed as a comparative framework rather than an experimental manipulation, and inferences about mechanisms are necessarily associative.

The sites, Wits Rural Facility (650 mm p.a.), Satara (550 mm p.a.) and Nwanedi Nature Reserve (380 mm p.a.) will hereafter be referred to by the rainfall they receive i.e., the 650 mm, 550 mm and 380 mm site.

### Experimental setup

We established four one-hectare plots in each of the three sites. Within the 550 mm site, plots were established within the unburned areas. Plots were spaced a minimum of 500 m apart. Decomposition assays and termite transects (100 m per plot) were undertaken on all twelve plots.

### Decomposition assays

Decomposition rate was estimated using decomposition bags and three substrates (grass, wood, and dung). *Themeda triandra* (Forssk.) was chosen as the grass because it has a moderate to high palatability, its distribution range encompassed all three sites and has previously been used in savanna decomposition experiments (e.g., Davies et al. 2013; Leitner et al. 2018). It was harvested from within and around the 650 mm site. After harvesting, grass material was cut into segments of approximately 5 cm, mixing stems, inflorescences, green and dried leaves. Pine (*Pinus radiata*) was selected as the wood substrate as it forms part of a global wood decomposition study (Zanne et al. 2022) allowing future cross-continental comparisons. Elephant (*Loxodonta africana*) dung was chosen due to its abundance in African savannas (elephant dung production is estimated to be 100 kg/elephant/day; Laws et al. 1975) and was collected in nearby game reserves in the Greater Kruger National Park.

Decomposition bags (20 × 20 cm) were constructed from 300-micron nylon mesh (Plastok) and closed with staples. Half of the bags had nine holes (8 mm diameter) punched on the underside of the bags allowing access by termites and other macroinvertebrates (**open bag** treatment). The second half of the bags were left intact, precluding access by termites and other macroinvertebrates (**closed bag** treatment). We used fine mesh bags with punched holes rather than coarse mesh bags to minimise leaching of substrates and to maintain similar microclimates in our two bag types. Grass bags contained 10 g of *T. triandra* oven-dried at 70 °C for 48 h. Wood decomposition bags each contained one wood block (9 cm x 9 cm x 5 cm block without bark, 405 cm^3^), which had been oven-dried at 120 °C for 48 h. Initial dry weight values were recorded for all wood blocks. Dung decomposition bags each contained 20 g of elephant dung oven-dried at 70 °C for 48 h. Each bag was pegged to the ground to secure it.

A total of 160 decomposition bags (80 wood, 40 grass and 40 dung) were placed in a four by five grid (10 m spacing between bags) on each plot (0.25 ha) at both the 650 mm and 380 mm site. While a total of 120 decomposition bags (80 wood and 40 grass) were placed in a four by five grid (10 m spacing between bags) on each plot (0.25 ha) at the 550 mm site. Dung decomposition bags were not set out at the 550 mm site owing to logistical constraints. All decomposition bags were set out at all three sites in December 2018 (Figure S2) at the onset of the rainy season (December – March across all three sites). Five open and five closed grass and dung litterbags were sacrificially removed from each plot at four-time intervals (14, 28, 56 and 112 days). Twenty open and twenty closed wood decomposition bags were sacrificially removed from each plot after 6 and 12 months.

After harvesting, the contents of the decomposition bags were removed. The remaining material was oven-dried at 80 °C for 72 h before cooling to room temperature. After drying, the original substrate was manually separated from any foreign plant material, soil or gallery material taken into the bags by termites. The bait substrate was then weighed to determine the proportional mass lost.

### The agents of decomposition

Both bag types (open and closed) allowed microarthropods (< 300 microns) and free-living microbes access to the substrate. The role of microarthropods in decomposition is difficult to establish as it involves teasing apart the effects of mesh size, substrate, microarthropod activity and microbial activity (Kampichler and Bruckner 2009). However, a review of previous microarthropod studies suggests that their contribution is negligible particularly outside of temperate humid zones (Kampichler and Bruckner 2009). A mesh size of 300-micron is small enough to exclude the larger microarthropods. In addition, because our bags were in the field from the start of the summer wet season when microbial decomposition would be highest, we assumed that the majority of decomposition that occurs in the closed bag treatment is by microbes.

The open decomposition bags also allowed access by termites and other macroinvertebrates. However, we assume that the greater part of non-microbial decomposition is termite-mediated because many studies have demonstrated that termites are the dominant macroinvertebrate decomposer of wood, dead grass and dry dung in African savannas (e.g., Buxton 1981; Collins 1981; Ouédraogo et al. 2004; Freymann et al. 2008; Veldhuis et al. 2017; Leitner et al. 2018). Fungus-growing termites, the dominant termite group in African savanna systems (Aanen and Eggleton 2005), typically construct runways and galleries from soil (sheeting) to cover both organic matter and their pathways to it. These soil structures protect the termites from desiccation and predation (Wood and Sands 1978). Following Veldhuis et al. (2017), we considered the presence of this sheeting in the open bags and also soil within the substrates, an indication of termite activity as no other animal produces sheeting and moves soil this way. To demonstrate the contribution of termites in our three study sites we assessed whether higher levels of substrate mass loss were linked to larger quantities of termite-moved soil. If higher levels of mass loss were associated with the presence of termite-moved soil, then we assumed the mass loss from open bags to be a combination of only microbial and termite decay.

### Termite community composition

We used the active-searching transect method (Davies et al. 2013) to describe the termite community at the wettest and intermediate site (650 mm and 550 mm). At our driest site (380 mm), we used a cellulose bait method which is considered more effective under those conditions (see Davies et al. 2021), however we also conducted active searching transects to enable direct comparisons of termite encounter rates among sites. Using the active searching transect method we searched for termites within each plot along a transect measuring 100 m x 2 m, subdivided into 20 contiguous sections each of 5 m x 2 m. Ten minutes of sampling effort was spent in each section, giving a total of 100 min sampling effort for each transect. All microhabitats in which termites are known to occur were searched, including logs and twigs, dung, tree trunks up to a height of 2 m, termite mounds, soil beneath logs, the base of grass clumps and the base of trees. Specifically, each encounter with a population of termites was recorded and a sample of workers, and where possible, soldiers were collected. Termites transects were undertaken in February 2019.

The cellulose bait method (following Davies et al. 2013; Davies et al. 2021) involved pegging sixteen toilet rolls (unbleached, single ply, unscented) to the ground, five meters apart, in a grid of four-by-four, across each plot. After 28 days, baits were inspected for termites and, where present, termite specimens were collected. Termite specimens were collected in February 2019. Both worker and soldier castes were collected where possible. Termites were identified at the University of Pretoria and the Natural History Museum, London (by KB and PE). Specimens were identified to genus, where possible, using soldier castes whenever available (when soldiers were not present, workers were used). Voucher specimens were lodged at the University of Pretoria, South Africa.

### Data analysis

#### Decomposition along a rainfall gradient

For each substrate, we assessed the effect of rainfall (380 mm, 550 mm and 650 mm savanna sites) and bag treatment (open and closed) on decomposition in terms of mass loss. We used the final collection time for wood (1 year), dung and grass (112 days) allowing ample time for the substrate to be found and decomposed. We used a linear mixed effects model with the proportion of mass loss as our response variable. Mass loss was logit transformed as is advised for proportional data (Warton and Hui 2011). Site (650 mm, 550 mm and 380 mm) and bag type (open and closed) were the explanatory variables and plot was used as the random factor. The model structure was as follows: proportional_massLoss ~ rainfall x bag type + (1|plot). Significant differences between the bag type and site were assessed using pairwise mean comparisons (Tukey’s posthoc tests) from the glht function in the ‘multcomp’ package in R version 3.4.3 (Hothorn et al. 2008).

### Invertebrate effects

To examine the contribution of termites across our substrates, we determined whether mass loss was correlated with the mass of termite sheeting within each decomposition bag. We regressed mass loss for wood (1 year), dung (112 days) and grass (112 days) against the mass of termite sheeting (grams). Mass loss was the response variable and termite sheeting mass, the explanatory variable. Separate regressions were performed for each site (650 mm, 550 mm, and 380 mm). In addition, we modelled this relationship using a mixed generalised linear model. The presence or absence of sheeting per bag was the binomial response variable, percentage mass loss was the fixed effect, and the random variable was plot.

### Percentage of decomposition performed by invertebrates and microbes

To establish the percentage of each substrate (wood, dung, and grass) that was decomposed by invertebrates across the three sites (650 mm, 550 mm, and 380 mm), we calculated the difference in mean mass loss between open and closed decomposition bag pairs. That is, the invertebrate contribution = mean mass loss in open bags – mean mass loss in closed bags. We attributed the percentage of mean mass loss in closed decomposition bags to microbes.

In addition, we calculated the relative contribution of macroinvertebrates versus microbes to the decomposition of each substrate across the study sites. To achieve this, we used the invertebrate contribution (%) and microbe contribution (%) and expressed these as a percentage of total decomposition.

### Decomposition rate

To determine how the decomposition rate varied across the rainfall gradient, we calculated the decomposition rate of each substrate at all three sites: 380 mm, 550 mm, and 650 mm. Following Olson (1963), we calculated the decomposition constant (k) and half-life (T_1/2_) for each site and bag type (open and closed) combination across all collection time points for each substrate (wood: 6 months and 1 year; dung and grass: 14, 28, 56 and 112 days) We calculated k using the equation below:

1 $$k = \frac{- \, \mathrm{natural} \, \mathrm{log}\, (X/X_0)}{t}$$

Half-life was calculated as below:

2 $$T_{1/2} = \frac{\mathrm{natural} \, \mathrm{log}\, (2)}{k}$$

where *t* is the time in years since the decomposition bag was placed on the plot, X is the substrate mass remaining at time point *t*, and X_0_ is the original mass at t = 0 years. This method assumes that *k* is constant. It is likely that *k* will change across the different collection time points due to seasonal effects. However, it is useful for estimating an average decomposition rate and as a means of comparing between substrates and treatments.

## Results

### Termites as dominant macroinvertebrates

In the macroinvertebrate-accessible (open) bags, termite sheeting and in-filled soil was found in 37.2% of wood, 38.0% of dung and 29.3% of grass decomposition bags (latest collection period). Across the three rainfall sites the presence of soil and therefore presence of termites, was linked with higher levels of mass loss (Fig. 3b). There is a significant positive relationship (Figure S3a) between mass loss from open decomposition bags and the presence of termite soil – wood (r-squared: 0.77–0.88), dung (r-squared: 0.64–0.65) and grass (r-squared: 0.64–0.80). Bags with the highest mass loss always contained soil (Figure S3b), showing that there is a strong correlation between soil and mass loss, which suggests that termites have an important role as decomposers of all three substrates.

### Decomposition along a rainfall gradient

A total of 1431 undisturbed decomposition bags were collected across all three substrates (wood, dung, and grass), time intervals, and sites (650 mm, 550 mm, and 380 mm). Dung decomposed the fastest, followed by grass, with wood decomposing the slowest (Table S4). Across all substrates and sites, open dung bags at the 650 mm site decomposed the fastest, with a half-life of 0.2 years (73 days). The greatest variation in decomposition rates was observed for wood: at the 380 mm site, open wood decomposition bags decomposed rapidly (k = 1.79), whereas at the 650 mm site, they decomposed very slowly (k = 0.33).

**Fig. 1:**
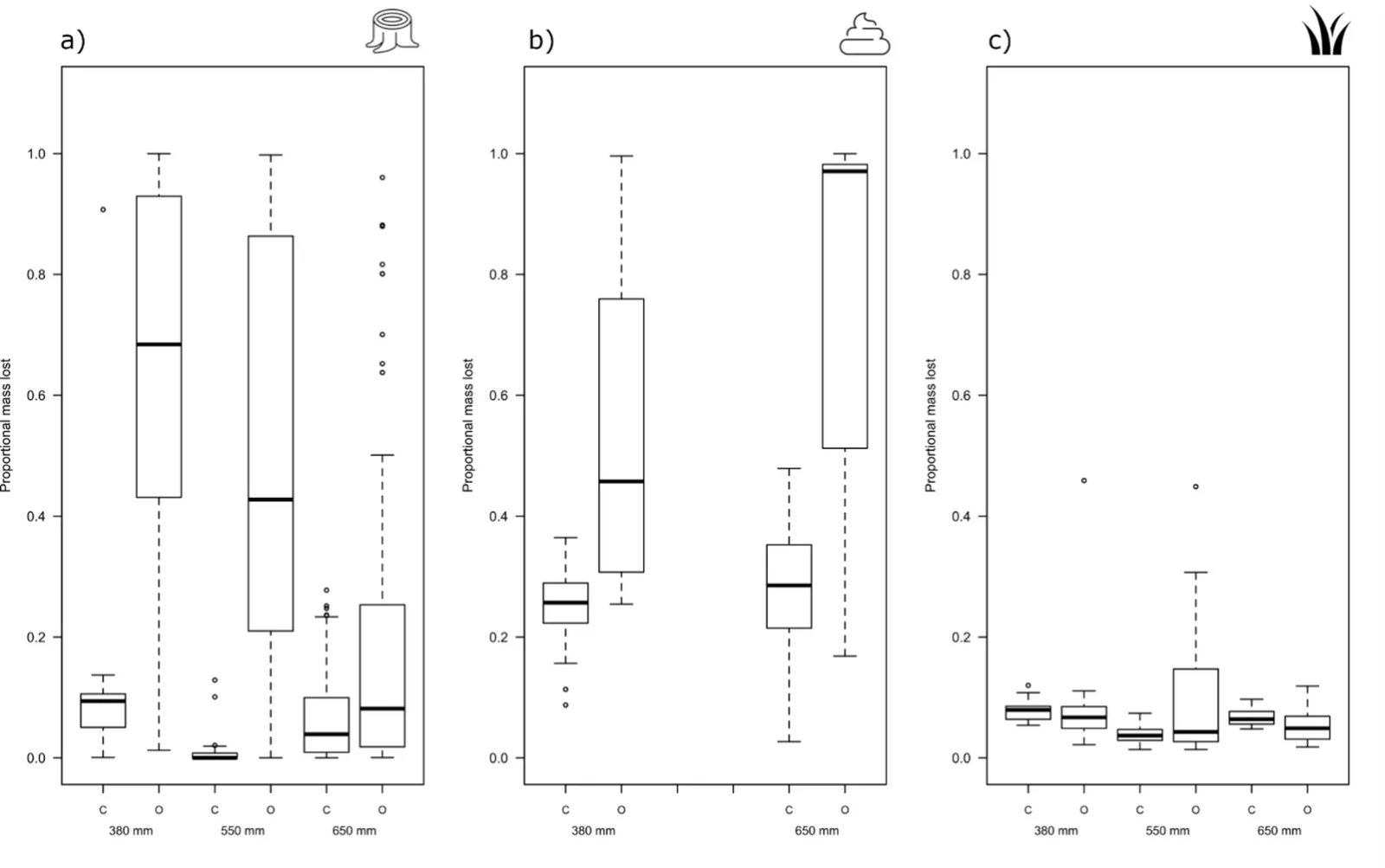
The median plus interquartile range for mass lost from closed (-C) and open (-O) decomposition bags [(**a**) wood 1 year; (**b**) dung 112 days and (**c**) grass 112 days] in the 380 mm, 550 mm, and 650 mm savanna sites. Horizontal lines at the top of the plots indicate significance of pairwise comparisons (Tukey test results (z-values): *p* < 0.05): light grey lines for intra-site significance between open and closed bags; solid lines for inter-site significance between closed bags and a dashed line for inter-site significance between open bags

**Fig. 2:**
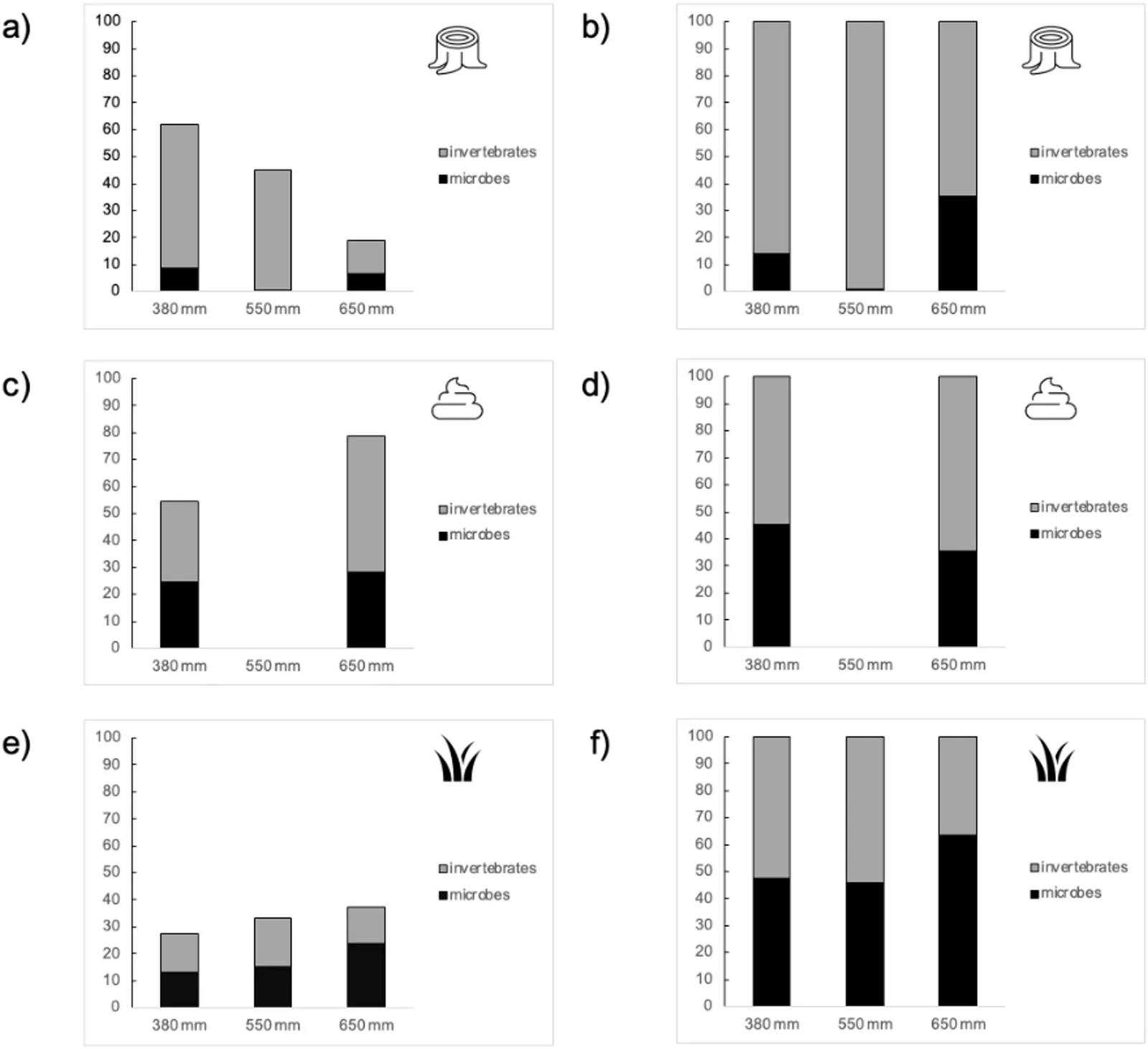
The percentage (left, **a**,** c & e**) and relative contributions (right, **b**,** d & f**) of microbes and termites to decomposition [(**a**) and (**b**) wood 1 year; (**c**) and (**d**) dung 112 days and (**e**) and (**f**) grass 112 days] at the 380 mm, 550 mm, and 650 mm savanna sites. The absolute contribution of microbes and termites to decomposition was calculated by comparing the percentage mean mass loss between open and closed decomposition bag pairs (Table S6). The relative contribution of each agent is the absolute decomposition value (Table S6) taken as a percentage of the total decomposition (mass lost from open decomposition bags) at each site

Wood decomposition decreased with increasing rainfall. A linear mixed-effects model revealed a significant site × bag type interaction (Table S1), justifying post hoc comparisons (Table S5). In open wood decomposition bags (decomposition by termites and microbes), mean mass loss was over three times higher at the 380 mm site (62.1%) than at the 650 mm site (18.9%; z = − 4.90, *p* < 0.001; Fig. 1). Intermediate decomposition occurred at the 550 mm site (45.1%). Termites accounted for 53.3% of absolute wood mass loss (1-year) at the driest site but only 12.2% at the wettest site (Fig. 2, Table S1). When comparing relative contributions, termites dominated wood decomposition across all three sites—accounting for 86% of total mass loss at the 380 mm site (vs. 14% microbes), and 65% at the 650 mm site (vs. 35% microbes; Fig. 2, Table S4). Both the absolute and relative contributions of termites were greatest at the lowest rainfall site.

In contrast to wood, dung and grass decomposition increased with rainfall. Mixed-effects models revealed significant interactions between site and bag type for both substrates (Table S2 for dung, Table S3 for grass), again justifying post hoc comparisons (Table S4). Mean mass loss from open dung and grass bags was significantly higher at the 650 mm site (78.9% dung, 37.4% grass) than at the 380 mm site (54.4% dung, 27.6% grass; z = 3.58 for dung, z = 2.624 for grass; *p* = 0.002 and 0.091 respectively; Fig. 1). Termites contributed the majority of absolute dung and grass mass loss at both sites (650 mm: 51% dung, 14% grass; 380 mm: 30% dung, 13% grass; Fig. 2, Table S4). For dung, termites accounted for a slightly larger proportion at the 650 mm site (64% termites vs. 36% microbes) than at the 380 mm site (55% vs. 45%). In contrast, for grass, microbes accounted for a greater share at the 650 mm site (63% microbes vs. 37% termites), while contributions were nearly equal at the 380 mm site (48% microbes vs. 52% termites; Fig. 2, Table S4).

Across all three substrates, mass loss was significantly greater in open bags (termites + microbes) than in closed bags (microbes only), consistent with a strong termite contribution to decomposition (Figs. 1 and 2, Table S4).

### Termite community composition

**Fig. 3:**
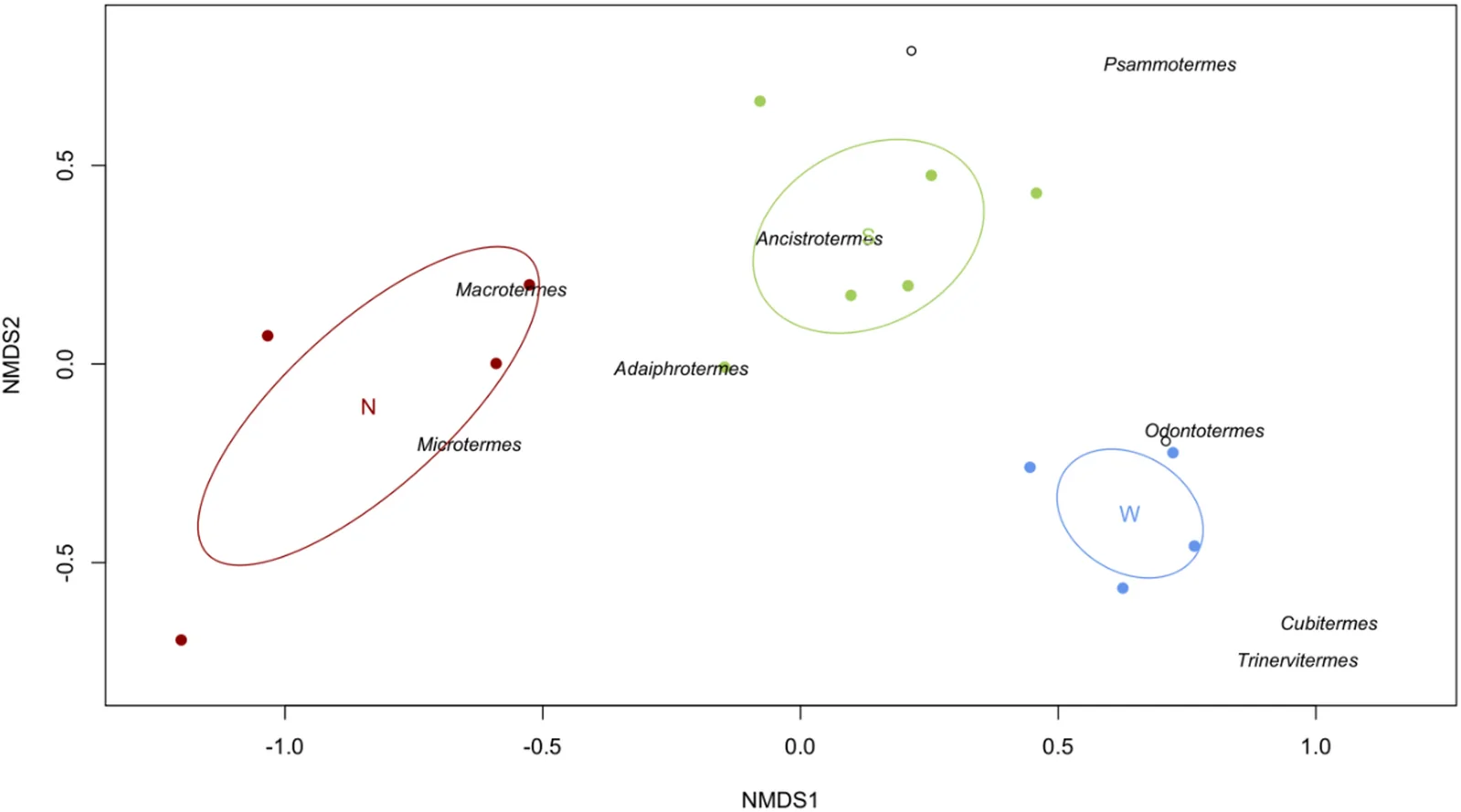
Non-metric multidimensional scaling (NMDS) ordination of termite community composition across three savanna sites differing in mean annual rainfall (380 mm, 550 mm, and 650 mm). Each point represents the termite community from one 1-ha plot, with colours indicating rainfall site: dark red (380 mm), light green (550 mm), and blue (650 mm). Termites were sampled using the active searching transect method (100 m transect per plot). Genus names are overlaid in black. Ellipses represent one standard deviation around the group centroid for each rainfall site, providing a visual summary of community variation within sites. Differences in community composition across sites were significant (ANOSIM: *R* = 0.86, *p* < 0.001). See Table S5 for the names of termite genera recorded across the three sites

**Fig. 4:**
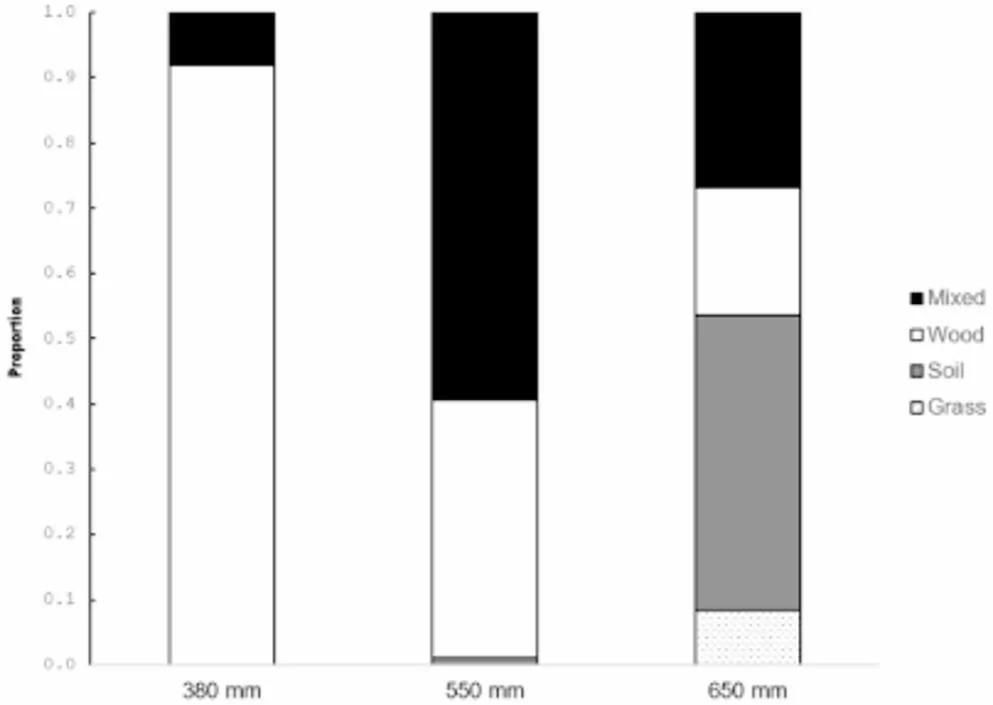
The proportion of termites encountered across feeding groups (Table S5) in the 380 mm, 550 mm and 650 mm savanna sites. The termites in the 380 mm site were sampled using the cellulose bait method while the termites in the 550- and 650-mm site were sampled using the active searching transect method

There were clear differences in the termite community composition among our sites (ANOSIM: *R* = 0.86, *p* < 0.001): Fig. 3). The termite community at the 380 mm site was dominated by wood-feeding termite specialists (Fig. 4). At the 550 mm site the termite community was predominantly composed of wood and mixed-feeding termites, while at the 650 mm site the termite community has an approximately equal representation across all feeding groups (mixed, soil, grass, and wood; Fig. 4). Only four termite genera occurred at the 380 mm site and 90% of all termite encounters were *Microtermes* (Family: Termitidae; Subfamily: Macrotermitinae, Table S5). In contrast, eight genera of termites occurred at the 550 mm site and nine at the 650 mm site (Table S5). At the 550 mm site, 70% of all encounters were with *Ancistrotermes* whereas at the 650 mm site none of the genera made up more than 50% of the termite community. *Nitiditermes* (formerly *Cubitermes* - soil-feeder) and *Ancistrotermes* (mixed-feeder) were the two termite genera encountered most often.

## Discussion

Global studies have demonstrated that decomposition is exceedingly climate-sensitive and driven by the complex interplay between temperature and precipitation (e.g. Seibold et al. 2021; Zanne et al. 2022). As temperature was relatively constant across our three study sites, we explored both the absolute and relative contribution of termites and microbes to changes in precipitation. Our study also examined the decomposition of multiple substrates and included dung, which has, to date, attracted little attention (Freymann et al. 2008). Published savanna decomposition studies vary considerably in substrate type, rainfall range, and whether termite contributions are explicitly quantified (Supplementary Table S9).

Contrary to expectation, wood decomposition decreased with increasing rainfall, driven by a reduced termite contribution. The absolute contribution of microbes to wood decomposition remained unchanged across the precipitation gradient. In contrast, both dry dung and grass decomposition increased with rainfall: dung decomposition rose due to greater termite contribution, whereas grass decomposition increased due to higher microbial activity. Thus, our prediction that decomposition would increase with rainfall held for dung and grass, but not wood. Termite decomposition also varied across the gradient, contrary to expectations.

However, the distinction between microbial and faunal contributions—based on closed versus open litterbags—should be interpreted with caution. Termite activity can stimulate microbial decomposition via fragmentation and aeration. Thus, the “faunal effect” likely includes both direct and indirect influences, and microbial contributions in closed bags may underestimate microbial activity under natural conditions. These interactions highlight that microbial and faunal processes are not independent but synergistic, representing an important conceptual limitation of this partitioning approach.

### Why agents responded as they did for the different substrates

#### Dung and grass

Dung decomposed faster than grass or wood. This underscores the role of termites in dry dung decomposition. Despite a large body of work on dung beetles, studies on termite-driven dung decomposition remain rare (Freymann et al. 2008; Hanski and Cambefort 1991). Rapid dung decomposition may be due to prior fragmentation and biochemical processing by herbivores and their gut microbes (Freymann et al. 2008).

We predicted that the relative contribution of microbes would increase with rainfall. In areas that experience high temperatures (such as our three study sites) soil moisture plays an important role in facilitating high rates of microbial activity. As a result, we expected that the rate of decomposition would rise with both increasing temperature and moisture (Peterjohn et al. 1994); this held for grass but in the case of dry dung, microbial decomposition remained relatively constant across the rainfall gradient.

The reason for this might be that microbial communities are specialised for local litter (home-field advantage) thus accelerating the rate of decomposition of grass relative to dung (Ayres et al. 2009; Palozzi and Lindo 2018; Lin et al. 2020). With increasing rainfall along the gradient, grass becomes a more reliable input whereas dung remains an irregular input. Thus, the microbial communities at our 550- and 650-mm sites might be more specialised for grass rather than dung decomposition. Alternatively, microbial activity across all our sites might be limited by water availability as all receive less than 700 mm per annum – roughly the point at which savannas switch from being water-limited or ‘climate-dependent’ ecosystems (200–700 mm p.a. Sankaran et al. 2008; Bond et al. 2003) to ‘disturbance-dependent’ ecosystems across a gradient of rainfall.

There is likely to have been a small leaching effect from dung bags. However, as the mesh was very fine, and as elephant dung is very coarse (large nonruminant herbivore, Owen-Smith 1982) leaching was assumed to be minimal. While it is possible that some photodegradation may have occurred within the dung and grass decomposition bags, since the top panels of both bag types were identical in design, all bags were subject to a similar degree of photodegradation and as a consequence our results will not be biased. In addition, the fine mesh of the decomposition bags is likely to offer the substrates some shade from radiation, so we expect photodegradation to be low (in keeping with Acanakwo et al. 2019). Notably, dung and grass bags were deployed during the rainy season, when both microbial and termite activity peak—so k-values likely represent upper decomposition limits.

#### Wood

The unexpected results we found for wood decomposition are puzzling. Not only did wood decomposition decrease with rainfall, contrary to expectation, but the rate of wood decomposition at our 650 mm site was also strikingly low (using Olson’s formula it is estimated to take over 100 years for wood to disappear; decomposition constant = 0.33, *Pinus radiata* wood density = 405 kg/m^3^ – a medium density ‘softwood’; Beets et al. 2007). The few other African studies that have calculated decomposition rates (k values) have all found the rate of wood disappearance to be far more rapid than that observed at our 650 mm site. Buxton (1981) working in the arid savannas of Tsavo East in Kenya (200–500 mm p.a.) found that wood disappeared within 8 to 11 years (decomposition constant = 0.10). Further, Collins (1977), working in the wet Southern Guinea savanna of Nigeria (1175 mm p.a.) - found a mean disappearance time for all wood of 2.0 years (decomposition constant = 0.49).

The decrease in wood decomposition with increasing rainfall is due to a reduction in the absolute and relative contribution of termites across the rainfall gradient. There are several possible explanations for this, some of which are not mutually exclusive. First, while there is a large overlap in the termite genera encountered at the three savanna sites (e.g., fungal-feeding termites, Macrotermitinae, occur at all three rainfall sites), there are marked differences in termite community composition by feeding group. Importantly, there is a decrease in both the absolute and the relative proportion of wood-feeding termites with an increase in rainfall (Fig. 4 and Figure S4).

A second possible explanation is that the termite community at the driest site is composed largely of subterranean, non-mound nesting termites. Subterranean termites have larger diffuse colonies made up of a vast network of interconnected fungal gardens (van de Peppel and Aanen 2020), which may mean that the termites associated with each fungal garden may be closer to food resources on the soil surface and therefore more likely to encounter the wood blocks. These termites use a combination of thermal shadows/differences in temperature and acoustic vibration (Evans et al. 2009) to locate these resources. In open habitats with bare ground, wood is likely to create a larger thermal shadow than would grass or dung. DeSouza et al. (2009) showed that the speed at which termites occupy cellulose baits was dependent on the size of the baits – the larger the bait, the more quickly it was occupied. The exploitation of a wood block might therefore represent a better investment of tunnel infrastructure than dung or grass. In contrast to termites with diffuse nest structures, mound-building termites are central place foragers (Turner 2019) with large colonies up to hundreds of meters apart. Once scouting termites have located a food resource on the soil surface, they then construct tunnels covered in soil sheeting to the food resource thus protecting themselves from desiccation and predation. A smaller food resource closer to the mound might be more efficient to exploit than a large food resource many tens of meters away. These differences in colony size and foraging strategy might underlie this puzzling pattern.

While these are potential explanations for the decline in termite wood decomposition with rainfall, they do not adequately account for the exceptionally low rate of wood decay at our highest rainfall site (650 mm; well under 20% consumption, k = 0.33) – less than a third of that of our dry site (380 mm; approx. 70%; k = 1.79) - and at the low end of the decay rates found for the subtropical regions in the global wood block study (Zanne et al. 2022). The termite community at the 650 mm site neither lacks species nor is density poor. Importantly, the number of termites encountered using the active searching transect method suggests that the density of termites increases with rainfall (Figure S4; Table S8). Furthermore, the number of termite encounters is in line with previous studies conducted in similar rainfall areas of the Kruger National Park (Davies et al. 2013) so it unlikely to be due to differences in the density of termites.

The unusually low rate of wood decomposition at the 650 mm site remains difficult to explain and likely results from a combination of interacting factors. One possible contributor is resource availability: the volume of coarse woody debris at the 650 mm site was more than seven times that observed at the 380 mm site (Figure S5). We also found that the termite discovery rate of experimental wood blocks was significantly lower at the 650 mm site than at the drier sites (Figure S6). This could suggest that high background wood availability dilutes encounter rates with new wood inputs. However, this remains speculative, and it is also possible that other factors—such as variation in wood-feeding termite abundance, or top-down control from predators such as ants—play a role. Notably, termite contributions to dung and grass decomposition were not similarly reduced at the 650 mm site, suggesting that any such controls may operate in a substrate-specific manner.

Consequently, the unusually low rate of wood decay at the wettest site (650 mm) is puzzling and impelled us to explore alternative explanations. Differences in coarse woody debris volume across sites point to resource availability as a possible driver: the 650 mm site contained more than seven times the dead-wood volume of the 380 mm site (Figure S5). In addition, termite discovery rates for experimental wood blocks were significantly lower at this site (Figure S6), suggesting that high background wood availability may dilute encounter rates with new wood inputs. We hypothesise that this reduced discovery reflects resource saturation, whereby the abundance of coarse woody debris lessens the likelihood that termites locate new resources. However, this remains speculative, and other ecological mechanisms may also contribute. Termite abundance was greatest at the 650 mm site, implying that coarse-wood accumulation does not result from termite scarcity per se. Instead, predation and intraspecific competition could constrain termite utilisation of available wood (Coggan et al. 2016). Indeed, experimental suppression of ant activity at this site has been shown to increase termite activity and elevate decomposition rates by 74–98% (Walker et al. 2022), indicating strong top-down control by ants. Notably, termite contributions to dung and grass decomposition were not similarly reduced at the wettest site, suggesting that these processes are influenced by different controls.

Decomposition studies rarely quantify the availability of resources within the study sites in which they are being performed (with the notable exception of Collins 1977 and Buxton 1981; who measured the standing crop of dead wood on the ground). Our findings highlight the considerable value of incorporating measures of resource availability and discovery rate in future work. Until these variables are routinely quantified, their relative importance in governing decomposition dynamics will remain poorly understood.

## Conclusion

Zanne et al.’s (2022) global decomposition study advances temperature as the most important climatic driver of decomposition. However, where temperature is relatively constant, such as across our study sites, our study revealed differences in precipitation within semi-arid savannas can strongly affect decomposition. We expected that the absolute decomposition of all three substrates would increase along the rainfall gradient. This hypothesis held for dung and grass, but we found the inverse for wood. Global decomposition studies (Seibold et al. 2021; Zanne et al. 2022), without exception, use a single substrate to measure decomposition. Our study underpins the importance of considering multiple substrates. In addition to observing an increase in wood decomposition as rainfall decreased, we also observed surprisingly low wood decomposition at our highest rainfall site. This prompted us to explore how much dead wood resource there was across our sites and how this affected termite discovery. There was almost an order of magnitude more coarse woody debris at our wettest site than at our driest site and this may have significantly reduced the rate of wood discovery by termites particularly given the low abundance of wood-feeders. Resource availability and termite discovery is an under-explored area in decomposition studies and is potentially critical to our understanding of decay dynamics. We suggest that resource availability is quantified in future decomposition studies.

## Supplementary Information

Below is the link to the electronic supplementary material.

Supplementary Material 1

## Data Availability

Data supporting the findings of this study are available from the corresponding author upon reasonable request.
