# Correction: Substrate type and termite discovery shape decomposition at the dry end of an African savanna rainfall gradient

**DOI:** 10.1007/s00442-026-05991-6

**Published:** 2026-10-15

**Authors:** Katherine Bunney, Mark Robertson, Paul Eggleton, Sally Archibald, Catherine Parr

**Affiliations:** 1 Department of Zoology & Entomology, University of Pretoria, Pretoria, South Africa; 2 Elephants Alive, Ekuthuleni Shareblock Ltd, Hoedspruit, South Africa; 3 Entomology Department, The Soil Biodiversity Group, The Natural History Museum, London, UK; 4 School of Animal, Plant and Environmental Sciences, University of Witwatersrand, Johannesburg, 2000 Republic of South Africa; 5 School of Environmental Sciences, University of Liverpool, Liverpool, UK

**Correction to: Oecologia (2026) 208:118**

https://doi.org/10.1007/s00442-026-05960-z

In the original version of this article, the first name and surname for author Sally Archibald have been rearranged which has now been replaced by the corrected version.

The original article has been corrected.

