# Supplementary material for: Substrate type and termite discovery shape decomposition at the dry end of an African savanna rainfall gradient
Source: Oecologia. 2026 Aug 28;208(9):118. doi: 10.1007/s00442-026-05960-z (PMC13525070; doi:10.1007/s00442-026-05960-z)
Supplement: Supplementary file 1 [file 442_2026_5960_MOESM1_ESM.docx]

**Figure S1a.** Rainfall across the three study sites during the decomposition experiment period (December 2018 to December 2019). Total cumulative rainfall for the 2019 period was: Nwanedi (380 mm site): 256 mm; Satara (550 mm site): 588 mm; and Wits Rural Facility (650 mm site): 745 mm. Monthly rainfall totals were recorded from on-site weather stations at each site.

**Figure S1b.** The mean minimum (blue) and maximum (red) monthly temperatures at the 380 mm, 550 mm and 650 mm savanna sites. Data derived from WorldClim (2021) based on site co-ordinates.

**Figure S2**: Layout of decomposition bags across the 50 m x 50 m (0.25 ha) plots. At each of the 20 positions (indicated by black squares), in the 4 x 5 grid, there were 8 decomposition bags. Four wood (W) - two open (O) and two closed (C); two grass (G) – one open (O) and one closed (C) and two dung (D) - one open (O) and one closed (C).

**Figure S3a.** The relationship between the presence of sheeting in open bags and mass loss (%) within open decomposition bags containing wood, grass and dung across all three sites, where 0 represents the absence of sheeting and 1 represents the presence.

**Figure S3b.** Mass loss (g) versus the quantity of termite soil (g) found within (a) wood 1 year; b) dung 112 days and c) grass 112 days open decomposition bags in the 380 mm (green), 550 mm (grey) and 650 mm (blue) savanna sites. The r-squared values appear brackets in legends of each plot.

**Figure S4.** (a) Absolute number and (b) relative proportion of termite encounters across feeding groups at the 380 mm, 550 mm and 650 mm savanna sites. Numbers within the black sections of panel (a) indicate the number of wood-feeding termite encounters. Termites were sampled using the active-searching transect method.

**Figure S5.** Histograms of coarse wood volume across a) 650 mm site and b) 380 mm site. The medians (a) 33.9 & b) 4.7 a indicated by vertical blue lines.

**Figure S6:** Proportion of wood decomposition bags discovered across the collection intervals (6 months (06m) & 12 months (12m)) at the 380 mm and 650 mm savanna sites.

**Table S1.** Fixed effects from a linear mixed-effects model of wood decomposition after 12 months.

Results from a linear mixed-effects model of wood decomposition after 12 months. R²m = 0.48, R²c = 0.55.

| **Fixed effect** | **df** | **Estimate** | **Standard error** | **t-value** | **p-value** |
| --- | --- | --- | --- | --- | --- |
| (Intercept: N.closed) | 8.17 | −2.788 | 0.480 | −5.805 | 0.0004 *** |
| Site: S | 13.65 | −4.188 | 0.705 | −5.943 | < 0.0001 *** |
| Site: W | 7.97 | −0.724 | 0.675 | −1.073 | 0.3148 |
| Bag type: open | 345.29 | 3.648 | 0.343 | 10.626 | < 0.0001 *** |
| Site S × bag open | 345.91 | 3.307 | 0.652 | 5.070 | < 0.0001 *** |
| Site W × bag open | 345.13 | −2.552 | 0.482 | −5.298 | < 0.0001 *** |

Interaction significance: Site × bag type: F(2, 345.56) = 43.02, p < 0.0001.

**Table S2.** Fixed effects from a linear mixed-effects model of dung decomposition.

The model includes only the N and W sites because data were unavailable for the 550 mm site. R²m = R²c = 0.43. A boundary singular-fit warning was produced because variance at the plot level was zero.

| **Fixed effect** | **df** | **Estimate** | **Standard error** | **t-value** | **p-value** |
| --- | --- | --- | --- | --- | --- |
| (Intercept: N.closed) | 75 | −1.169 | 0.439 | −2.664 | 0.0094 ** |
| Site: W | 75 | 0.111 | 0.613 | 0.181 | 0.8567 |
| Bag type: open | 75 | 1.892 | 0.613 | 3.088 | 0.0028 ** |
| Site W × bag open | 75 | 2.057 | 0.861 | 2.389 | 0.0194 * |

Interaction significance: Site × bag type: F(1, 75) = 5.71, p = 0.019.

**Table S3.** Fixed effects from a linear mixed-effects model of grass decomposition.

R²m = 0.14, R²c = 0.35.

| **Fixed effect** | **df** | **Estimate** | **Standard error** | **t-value** | **p-value** |
| --- | --- | --- | --- | --- | --- |
| (Intercept: N.closed) | 14.15 | −2.490 | 0.214 | −11.624 | < 0.0001 *** |
| Site: S | 17.00 | −0.801 | 0.275 | −2.916 | 0.0096 ** |
| Site: W | 14.45 | −0.174 | 0.305 | −0.572 | 0.5761 |
| Bag type: open | 99.97 | −0.111 | 0.189 | −0.585 | 0.5598 |
| Site S × bag open | 100.24 | 0.671 | 0.260 | 2.579 | 0.0114 * |
| Site W × bag open | 100.07 | −0.220 | 0.275 | −0.799 | 0.4264 |

Interaction significance: Site × bag type: F(2, 100.22) = 6.23, p = 0.0028.

**Table S4.** Post hoc pairwise comparisons of decomposition rates across rainfall sites and bag treatments.

Z-values from Tukey’s post hoc tests comparing decomposition rates by site and bag type (closed = microbes only; open = microbes + termites). Blank cells indicate comparisons that were not applicable. Significance codes: p < 0.001 (***); p < 0.01 (**); p < 0.05 (*).

| **Comparison** | **Wood** | **Dung** | **Grass** |
| --- | --- | --- | --- |
| **Closed vs closed (microbial only)** |  |  |  |
| 380 mm – 550 mm | −3.52 (**) |  |  |
| 550 mm – 650 mm | −3.28 (***) |  | 3.59 (**) |
| 380 mm – 650 mm |  |  |  |
| **Open vs open (microbial + termites)** |  |  |  |
| 380 mm – 550 mm |  |  |  |
| 550 mm – 650 mm |  |  | 3.50 (**) |
| 380 mm – 650 mm | −3.17 (*) | 3.58 (**) | 2.86 (*) |
| **Open vs closed (same site)** |  |  |  |
| 380 mm | 14.60 (***) | 3.08 (*) | 3.71 (**) |
| 550 mm | 13.95 (***) | — | — |
| 650 mm | 3.31 (**) | 6.53 (***) | 3.09 (*) |

**Table S5.** Termite genera recorded across the three savanna sites (380 mm, 550 mm and 650 mm) and their feeding groups.

| **Termite genus** | **Feeding group (Uys 2002)** | **Donovan feeding group (Donovan et al. 2000, 2001)** | **380 mm** | **550 mm** | **650 mm** |
| --- | --- | --- | --- | --- | --- |
| *Adaiphrotermes* | soil | III |  | X |  |
| *Allodontermes* | mixed | IIf |  | X | X |
| *Ancistrotermes* | mixed | IIf | X | X | X |
| *Angulitermes* | dung | IIf |  |  | X |
| *Nitiditermes* | soil | IV |  |  | X |
| *Macrotermes* | mixed | IIf | X | X | X |
| *Microcerotermes* | wood | IIf |  | X |  |
| *Microtermes* | wood | IIf | X | X | X |
| *Odontotermes* | wood | IIf |  | X | X |
| *Psammotermes* | wood | I |  | X |  |
| *Schedarhinotermes* | grass | I |  |  | X |
| *Trinervitermes* | grass | I |  |  | X |
| *Unguitermes* | soil | IV | X |  |  |

Group I: lower termites feeding on dead wood and grass. Group II: Termitidae with a range of feeding habits, including dead wood, grass, leaf litter and micro-epiphytes. Group III: Termitidae feeding in the organic-rich upper layers of the soil. Group IV: true soil-feeding Termitidae that ingest apparently mineral soil.

**Table S6.** Mean wood, dung and grass mass loss (g) attributed to microbes and invertebrates at the 380 mm, 550 mm and 650 mm savanna sites.

| **Substrate** | **Biological agent** | **Calculation** | **380 mm** | **550 mm** | **650 mm** |
| --- | --- | --- | --- | --- | --- |
| Wood, 1 year | Microbes | Closed bag | 8.81 | 0.43 | 6.65 |
| Wood, 1 year | Invertebrates | Open bag − closed bag | 53.32 | 44.65 | 12.20 |
| Dung, 112 days | Microbes | Closed bag | 24.64 | NA | 28.12 |
| Dung, 112 days | Invertebrates | Open bag − closed bag | 29.71 | NA | 50.75 |
| Grass, 112 days | Microbes | Closed bag | 13.16 | 15.19 | 23.75 |
| Grass, 112 days | Invertebrates | Open bag − closed bag | 14.44 | 17.93 | 13.68 |

**Table S7.** Decomposition constants (k) and half-lives (in brackets) for decomposition bags containing wood, dung and grass across all collection intervals at the two ends of the decomposition gradient (380 mm and 650 mm).

| **Treatment** | **Biological agent** | **380 mm** | **650 mm** |
| --- | --- | --- | --- |
| Wood open | Invertebrates + microbes | 0.3 (2.54 years) | 0.006 (108.12 years) |
| Wood closed | Microbes | 0.02 (29.23 years) | 0.005 (145.30 years) |
| Dung open | Invertebrates + microbes | 2.47 (0.28 years) | 3.46 (0.20 years) |
| Dung closed | Microbes | 1.38 (0.50 years) | 1.44 (0.48 years) |
| Grass open | Invertebrates + microbes | 0.42 (1.64 years) | 1.24 (0.56 years) |
| Grass closed | Microbes | 0.34 (2.05 years) | 1.2 (0.58 years) |

**Table S8.** Number of termite encounters per 100 m of transect at the 380 mm, 550 mm and 650 mm savanna sites.

| **Site** | **Mean per 100 m** | **Minimum per 100 m** | **Maximum per 100 m** |
| --- | --- | --- | --- |
| 380 mm site | 21.75 | 13 | 29 |
| 550 mm site | 31.2 | 25 | 40 |
| 650 mm site | 54.5 | 30 | 85 |

**Table S9.** Summary of published savanna decomposition studies across rainfall gradients and related termite-mediated decomposition studies referenced in this manuscript.

| Lead author | Year | Region / system | Rainfall range (mm p.a.) | Substrate(s) | Termite access quantified / isolated? | Key finding relevant here |
| --- | --- | --- | --- | --- | --- | --- |
| Buxton | 1981 | Kenya (Tsavo East NP) | 200–500 | Wood | Yes (termite contribution assessed) | Wood decomposition by termites increased with rainfall across semi-arid sites. |
| Collins | 1981 | Nigeria (Southern Guinea savanna) | 1175 | Wood and leaf litter | Yes (termite role assessed) | High termite involvement; rapid wood and litter decomposition in wet savanna. |
| Schuurman | 2006 | Botswana (semi-arid; fungus-growing termite-dominated assemblage) | Semi-arid (site-specific) | Multiple resources (foraging focus) | Indirect (community/foraging patterns) | Assemblage dominated by fungus-growing termites; foraging and distribution patterns relevant to discovery processes. |
| Freymann | 2008 | Tropical ecosystems (review) | Various | Dung | Varies (review) | Highlights the importance of termites in dung recycling and notes the limited number of empirical studies. |
| Veldhuis | 2017 | South Africa (Hluhluwe-iMfolozi Park) | 500–900 | Dung | No (termites and microbes not separated) | Dung decomposition decreased with rainfall; demonstrates rainfall-related variability in dung decay. |
| Davies | 2015 | South African savannas (multiple savanna types) | 450–900 | Grass | No | Grass decomposition increased under warmer and wetter conditions across savanna types. |
| Buitenwerf | 2011 | South Africa (Hluhluwe-iMfolozi Park) | 630–1000 | Grass (litter removal) | Yes (termite activity quantified) | Termite-mediated litter removal varied across the rainfall gradient, linking herbivores, termites and rainfall. |
| Gosling | 2016 | South Africa (Hluhluwe-iMfolozi Park) | 630–1000 | Grass (termite activity) | Indirect (activity measures) | Rainfall, grass height and herbivores drove termite activity; supports context dependence across gradients. |
| Leitner | 2018 | African savanna (woody-encroachment context) | Savanna (site-specific) | Wood / litter | Termite activity assessed | Woody encroachment slowed decomposition and termite activity; shows that substrate and resource context matter. |
| Ouédraogo | 2004 | Semi-arid West Africa | Semi-arid (site-specific) | Organic resources (multiple) | Yes (macrofauna-mediated disappearance) | Soil macrofauna, including termites, strongly mediated the disappearance of organic resources in semi-arid systems. |
| Acanakwo | 2019 | African savanna (termite mounds) | Savanna (site-specific) | Wood | Yes (mound versus off-mound) | Wood decomposition was faster on termite mounds than off mounds, demonstrating strong termite effects on wood decay. |

**Resource availability and termite discovery**

We explored resource availability and termite discovery to help interpret our findings.

**Methods**

**Resource availability**

To establish whether the rate of wood decomposition was related to the amount of coarse woody debris present on the soil surface, we conducted coarse woody debris transects at the two ends of the rainfall gradient—the 380 mm and 650 mm sites—in October 2019. We used a line-intercept method (see Marshall and others 2000), in which two 50 m tapes were laid across each plot and every piece of dead wood intersecting a tape was measured for length and diameter. Wood volume across each plot was calculated using the formula: pole length × π × diameter.

**Termite discovery**

We used the presence of termite sheeting to confirm discovery of individual decomposition bags. This allowed us to determine whether the rate of discovery across collection intervals differed among sites. We note that using soil as an indicator of termite activity provides a conservative estimate because it does not account for the activity of non-fungus-growing termites such as *Trinervitermes*, a grass-feeding specialist that does not produce sheeting.

**Results**

**Resource availability**

The highest-rainfall site (650 mm) had seven times the coarse wood volume of the driest site (380 mm; Figure S5). Median wood volume was 33.9 cm³ at the 650 mm site and 4.7 cm³ at the 380 mm site.

**Termite discovery**

Termites discovered significantly more wood decomposition bags at the 380 mm site than at the 650 mm site, both after 6 months and after 1 year. After 6 months, 85% of wood decomposition bags at the 380 mm site had been discovered, compared with 56% at the 650 mm site. There was greater variability in the proportion of bags discovered among plots at the 650 mm site (30–79%) than at the 380 mm site (63–100%).
